# The burden of HIV mortality following the rollout of antiretroviral therapy: evidence from an observational community cohort study in KwaZulu-Natal, South Africa

**DOI:** 10.1016/S2352-3018(16)30225-9

**Published:** 2016-12-10

**Authors:** G. Reniers, S. Blom, C. Calvert, A. Martin-Onraet, K. Herbst, J.W. Eaton, J. Bor, E. Slaymaker, Z.R. Li, S.J. Clark, T. Bärnighausen, B. Zaba, V. Hosegood

**Affiliations:** 1 Department of Population Health, London School of Hygiene and Tropical Medicine, London, United Kingdom; 2 School of Public Health, University of the Witwatersrand, Johannesburg, South Africa; 3 Instituto Nacional de Cancerología, Mexico City, Mexico; 4 Africa Health Research Institute, Durban, South Africa; 5 Department of Infectious Disease Epidemiology, Imperial College, London, United Kingdom; 6 Department of Global Health, Boston University, Boston, United States; 7 Department of Statistics, University of Washington, Seattle, United States; 8 Department of Sociology, The Ohio State University, Columbus, Ohio; 9 Institute of Public Health, University of Heidelberg, Heidelberg, Germany; 10 Harvard T.H. Chan School of Public Health, Harvard University, Boston, United States; 11 Social Statistics and Demography, University of Southampton, Southampton, United Kingdom

## Abstract

**Background:**

Antiretroviral therapy (ART) substantially decreases morbidity and mortality among people living with HIV. In this study, we describe population-level trends in the adult life expectancy (LE), and trends in the residual burden of HIV mortality following the rollout of a public sector ART programme in one of the populations with the most severe HIV epidemics in the world.

**Methods:**

Data come from a demographic and HIV surveillance system in northern KwaZulu-Natal (South Africa), and cover the calendar years 2001 through 2014. We use non-parametric survival analysis methods to estimate gains in the population-wide LE at age 15 since the introduction of ART, and the shortfall of the population-wide adult LE compared to that of the HIV negative population (i.e., the LE deficit). LE gains and deficits are further disaggregated by age and cause of death using demographic decomposition methods.

**Findings:**

The dataset contains information on 93,903 adults who jointly contribute 535,428 person-years of observation to the analyses and 9,992 deaths. Since the rollout of ART in 2004, adult LE increased by 15·2 years for men (95%-CI: 12·4-17·8), and 17·2 years for women (95%-CI: 14·5-20·2). Reductions in pulmonary TB and HIV related mortality account for 79·7% of the LE gains among men, and 90·7% among women. For men, 9·5% is the result of a decline in external injuries. By 2014, the LE deficit had contracted to 1·2 years for men (95%-CI: -2·9-5·8) and to 5·3 years for women (95%-CI: 2·6-7·8). Pulmonary TB and HIV are responsible for 84·9% of the LE deficit among men in 2011-'14, and for 80·8% among women.

**Interpretation:**

The burden of HIV on adult mortality in this population is rapidly shrinking, but remains sizable for women, despite their better engagement with HIV care services. The recent gains in adult life-years lived as well as the current LE deficit are almost exclusively due to differences in mortality attributed to HIV and pulmonary TB.

**Funding:**

Wellcome Trust, the Bill and Melinda Gates Foundation, and the National Institutes of Health.

## Introduction

The rollout of antiretroviral therapy (ART) in populations with generalized epidemics has greatly improved the survival of people living with HIV (PLHIV), and that has been documented in both clinical cohorts and population-based research.^1-7^ Many studies report on changes in all-cause mortality, but do not quantify how much of the overall mortality decline is due to a reduction in HIV associated mortality. In addition, most studies are not in a position to estimate the residual burden of HIV on population-level adult mortality. We seek to remedy both shortcomings with data from a demographic and HIV surveillance site in rural KwaZulu-Natal where HIV status is known for a large portion of the population.

We report on trends in the life expectancy (LE) at age 15, and the adult LE by HIV status. LE is one of the most widely used summary measures of mortality and well-suited to quantify the effects of ART because it values the prolongation of life in addition to the mere elimination of deaths from a particular cause. Other adult mortality measures, including the probability of dying in adulthood (_45_q_15_) are less sensitive to the shift in the age distribution of deaths and may therefore underestimate the mortality reductions prompted by the rollout of ART.

Our analyses focus on two quantities of great public health interest, namely the gains in adult LE since the introduction of ART, and trends in the LE deficit. The LE deficit is defined as the shortfall of the population-wide LE compared to that of the HIV negative population, and quantifies the residual burden of HIV mortality in a population. Further, we use demographic decomposition techniques to estimate the contribution of changes in HIV and other causes of death (CoD) to recent trends in the adult LE and the adult LE deficit. These analyses thus update and extend previous work on adult mortality from the same study site.^2,8-10^ All our estimates are disaggregated by sex, and add a new perspective to the literature wherein women are routinely considered to have disproportionally benefited from the expansion of treatment.^11,12^

## Methods

### Study design and population

Data come from the Africa Centre Demographic Information System (ACDIS) in the uMkhanyakude District in northern KwaZulu-Natal, covering 434km^2^ of predominantly rural area with a resident adult population of around 45,000 adults (ages 15 years and above).^13^ The population is characterized by high HIV prevalence (29% among adults aged 15-49 in 2011),^14^ high levels of cardiovascular risk factors, and high mortality from external injuries.^2^

The public sector ART programme in the study area enrolled its first patients in August 2004. By the end of 2006 over a thousand patients were receiving treatment, and by mid-2011 an estimated 37% of PLHIV in the study population were on ART. ^10,15^ More detail about the expansion of the treatment programme in South Africa, and changes to the ART eligibility criteria is given elsewhere.^16^

### Data collection

Demographic surveillance is conducted through tri-annual household visits, and population-based HIV testing of resident adults is done annually – since 2003-'04 for men and women of reproductive age, and since 2007 for all adults. HIV status information is also obtained through record linkage with health facilities providing ART in the area covered by the ACDIS.

Individuals contribute person-time to the analyses from their fifteenth birthday or from when they move into one of the villages under surveillance, and until they move out, die, or turn 100 years old. The data extraction from the ACDIS database was done in August 2015, and observations were administratively censored at the end of 2014.

In order to allocate person-time to HIV status categories we classify the time prior to the first recorded HIV test as HIV status unknown. The time following a positive test remains positive until censoring or death. The time following the last negative test is considered negative for a duration of five years, after which it is classified as unknown. This procedure allows for the estimation of mortality rates among HIV negative individuals, but the exposure time is sufficiently short to ensure that elevated mortality among seroconvertors does not introduce bias (appendix, pp. 2-3). Time between two HIV negative tests is always counted as negative no matter how long the interval between tests.

Ethical approvals for this study were obtained from the Biomedical Research Ethics Committee of the University of KwaZulu-Natal, and the Observational Research Ethics Committee of the London School of Hygiene and Tropical Medicine. Verbal informed consent was obtained from household representatives for the demographic surveillance, and individual written consent for the HIV surveillance. The analyses conducted for this paper consisted of secondary analysis of de-identified data. The core ACDIS dataset is publicly available through the INDEPTH Network Data Repository (INDEPTH.ZA031.CMD2014.v1).

### Statistical analysis

We present non-parametric estimates of adult LE by sex, year and HIV status. Adult LE is defined as the number of additional years that a survivor to age 15 can expect to live if subject to the mortality observed in a specific period (appendix, pp. 4-5). LE estimates are computed with continuous-time survival analysis techniques as the area under the Kaplan Meier survival curve. Percentile-based confidence bounds are obtained via bootstrapping with 1,000 replications.

The discussion below focuses on two quantities: (i) *the total adult LE gain* since introduction of ART, and (ii) the remaining *adult LE deficit*. The total LE gain is the difference between the LE estimates for 2003 and 2014, which represent the calendar year before ART became available at local health facilities and the last year with available data. The adult LE deficit quantifies the extent to which the overall population LE falls short of the LE of HIV negative individuals. In other words, it is a summary measure of the mortality impact of the HIV epidemic conditional on the background health profile of its population, and is directly affected by HIV epidemic severity and efforts to mitigate its mortality impact (e.g., ART).

Adult LE gains and deficits are disaggregated by age and cause of death using a demographic decomposition method first proposed by Arriaga.^17^ The decomposition of the LE gains quantifies the contribution of changes in cause-specific mortality in each age group to the overall increase in adult LE from 2000-'03 (the pre-ART period) to 2011-'14. The decomposition is done for groups of calendar years because the population is too small for an analysis by single year. The same methodology is subsequently used for decomposing the LE deficit for two periods (2007-'10 and 2011-'14). Their comparison informs us of changes in the age and cause distribution of excess mortality as treatment programs come to maturation.

Information on the CoDs comes from verbal autopsy (VA) interviews with relatives of deceased individuals. VA interpretation is done with the InSilicoVA tool.^18^ InSilicoVA uses a Bayesian model to estimate the cause-specific mortality fractions (CSMF) at the population level, and cause-specific probabilities at the individual level. We separately generate estimates for sub-populations defined by two broad age groups (below 60 and 60 or older), gender, and HIV status at the time of death.

We then aggregate the individual-level cause-specific probabilities to obtain CSMF estimates for five-year age groups. The CoD classification scheme for reporting results distinguishes HIV/AIDS, pulmonary tuberculosis (TB), other communicable diseases, malignant neoplasms, cardiovascular disease, other non-communicable diseases, external causes, and maternal mortality. We report results for pulmonary TB and HIV separately, but we know from prior work that they are often difficult to separate on the basis of a VA interview due to the similarity of symptoms and high co-morbidity.^19^ The online appendix maps the CoD classification scheme onto the ICD-10 (pp. 6), and summarizes analyses wherein the CoD attribution has been done with the InterVA model (pp. 7).^20^

### Role of the funding source

The funders played no role in study design, in the collection, analysis, or interpretation of data, in the writing of this report, or in the decision to submit for publication. The corresponding author (GR) had full access to all the data in the study and had final responsibility for the decision to submit for publication.

## Results

Between 2001 and 2014, a cumulative number of 93,903 adults ever resided in the demographic surveillance area. They jointly contribute 535,428 person-years of observation time and 9,992 deaths to the analyses (Table 1). Verbal autopsy interviews were completed for 9,605 of these deaths. Women contribute 59% of the person-years of exposure to the study, and the HIV status is known for around 36% of the total person-years lived. The online appendix (pp. 2-3) provides more detail on the HIV status information in the dataset.

The crude death rate decreased from 23·1 (95%-CI: 22·3-23·9) to 13·6 (95%-CI: 13·0-14·2) per 1,000 person-years between 2001-'04 and 2011-'14. Because HIV surveillance only commenced in 2004, mortality estimates by HIV status before the rollout of ART are not available. However, the most pronounced mortality declines took place between ages 20 and 45, which is an age range where HIV-associated mortality is common.

**Figure 1:**
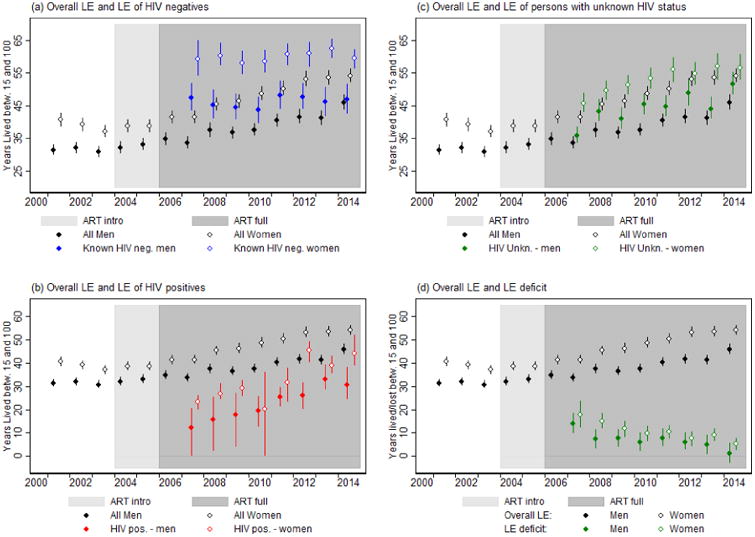
Adult LE trends by sex and HIV status, and the LE deficit (uMkhanyakude, 2001-'14)

The mortality rate reductions translate into important gains in adult LE (Figure 1 and pp. 4-5 in the appendix). Overall adult LE reached its nadir in 2003, the year before the introduction of ART. Between 2003 and 2014, adult LE increased by 15·2 years for men (95%-CI: 12·4-17·8), and 17·2 years for women (95%-CI: 14·5-20·2). These estimates represent average increases in the adult LE of 1·38 years per annum for men and 1·58 years for women, and are much larger than the LE gains estimated for South Africa as a whole (appendix, pp. 8). In 2014, the population-wide adult LE in uMkhanyakude reached 45·9 (95% CI: 43·7-48·4) and 54·2 (95% CI: 52·2-56·2) years for men and women, respectively.

Adult LE estimates by HIV status can be computed from 2007 onwards; the year that the HIV testing eligibility criteria were expanded to all adults. The adult LE for HIV negative men and women hovered around 47 years for men and 60 years for women for the entire period (Figure 1a). Whereas the adult LE of HIV negatives did not change, the outlook for PLHIV improved dramatically. Between 2007 and 2014, the number of years that an adult HIV positive person can expect to live increased by 18·4 (95% CI: 7·5-33·8) years for men and 20·7 (95% CI: 14·5-29·2) years for women (Figure 1b). By 2014, a 15-year old HIV positive man could expect to live for another 30·5 years (95%-CI: 24·4-38·3) at the prevailing mortality rates. For women this was 44·1 years (95%-CI: 39·1-52·1). The LE of adults whose HIV status is unknown to the study is marginally higher than that of the population as a whole, which suggests that PLHIV are under-represented among those with an unknown HIV status (Figure 1c).

The LE estimates reported in Figure 1a are the inputs for computing the LE deficit associated with HIV (Figure 1d). In 2007, three years after the introduction of ART at local health facilities, this deficit was still 13·8 years (95%-CI: 9·7-18·6) for men and 17·7 years (95%-CI: 12·3-23·8) for women. By 2014, the LE deficit reduced to 1·2 (95%-CI: -2·9-5·8) and 5·3 (95%-CI: 2·6-7·8) years for men and women, respectively.

**Figure 2:**
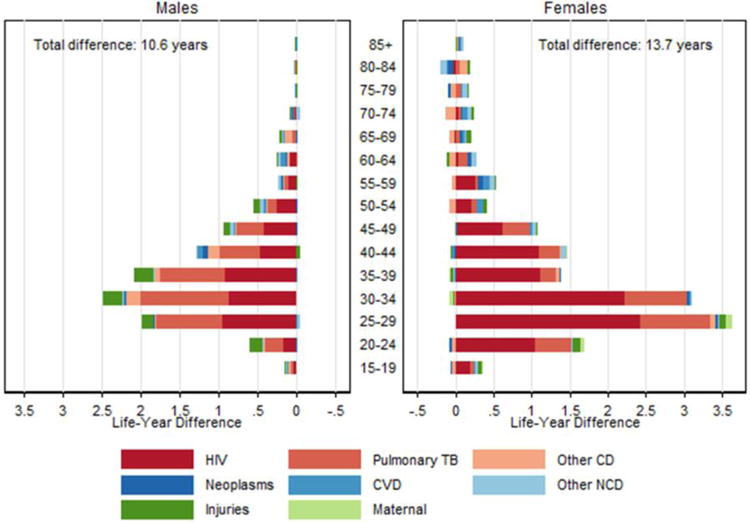
Age and cause-group contributions to the gross LE gains between 2001-'04 and 2011-'14, by sex (uMkhanyakude)

Figure 2 provides insight into the age groups and CoDs that have contributed to the LE gains since the introduction of ART. CoD contributions aggregated over age are reported in Table 2. The total LE gain that is decomposed in Figure 2 amounts to 10·6 years for men and 13·7 years for women, and pertains to the 10-year interval between 2001-'04 and 2011-'14. Negative values in these plots indicate that the mortality rates from a particular cause in a specific age group increased over time and thus had a negative effect on the LE trend. Their contributions are very small. The age groups with the largest contribution to the increase in adult LE over this period are 25-29 years for women, and 30-34 years for men.

The decomposition by cause indicates that almost all of the recent gains in adult LE result from reductions in mortality ascribed to HIV and pulmonary TB. Among men, these two causes alone account for a gain of 8·4 adult life-years, or, 79·7% of the total gain. The gain in adult LE ascribed to reductions in pulmonary TB and HIV mortality among women is 12·8 years, or, 90·7% of the total. The relative contribution of HIV within this group of causes is larger for women than for men. Among men, a reduction in the number of deaths from external injuries contributes one year to the increase in adult LE in the decade under consideration, which corresponds to 9·5% of the total LE gain. All the other CoD groups contribute less than one year to the change in the adult life-years lived for both men and women.

**Figure 3:**
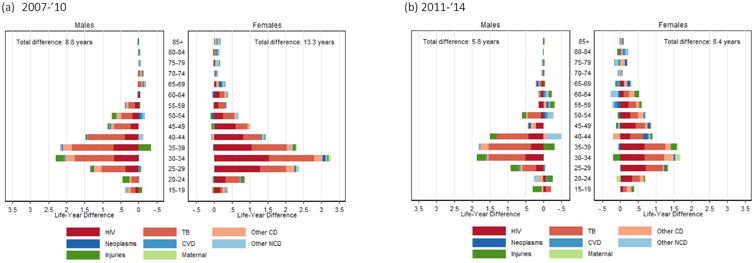
Age-cause decomposition of the LE deficit in 2007-'10 and 2011-'14, by sex (uMkhanyakude)

Figure 3 shifts the focus to the age-groups and CoDs that contribute to the shortfall in the population-wide LE compared to the HIV negative population. To elicit changes over time, the age-cause decomposition of the LE deficit is done for two periods, which indicates that the age profile of the LE deficit is becoming older. This is particularly the case for women, for whom the median of the age-group contributions to the LE deficit increased from 34·9 to 36·3 years between 2007-'10 and 2011-'14.

Pulmonary TB and HIV account for most of the shortfall in the population-wide adult LE in both periods (Table 2). Among men, they account for 85·0% of the LE deficit in 2007-'10 and 84·9% in 2011-'14. Among women, their contributions are 85·5% and 80·8%, respectively. Most of the remainder is attributed to other communicable diseases. The contributions of non-communicable diseases, external injuries and maternal causes to the LE deficit are small.

## Discussion

The introduction of ART in public sector health facilities in KwaZulu-Natal marked the starting point for unprecedented population-wide increases in adult LE of 1·38 and 1·58 years per annum for men and women, respectively. The total gains in adult LE between 2003 and 2014 amount to 15·2 years for men (95%-CI: 12·4-17·8) and 17·2 years for women (95%-CI: 14·5-20·2), and extend earlier estimates for this population.^8^ In comparison, the adult LE in Japan after World War II increased at an average rate of around 0·5 years per annum for a total gain of 9·4 years between 1947-'49 and 1965-'69, ^21^ and is one of the nations with the most rapid LE increases on record. The pace of the adult LE increases in this population in KwaZulu-Natal has been three times faster, and is almost exclusively driven by reductions in HIV related mortality. This conclusion is supported by concomitant increases in the adult LE of PLHIV, the lack of a decline in the mortality of HIV negatives, and an analysis of changes in the CoD structure. A decline in the deaths ascribed to pulmonary TB and HIV alone account for around 79·7 and 90·7% of the total LE gain for men and women, respectively. Among men, a decline in mortality from external injuries represents an additional 9·5% of the adult LE gain in the decade following the rollout of ART.

The LE gains directly attributable to ART are probably even larger than the observed increases in adult LE because mortality trends also depend on historical patterns of HIV incidence. The HIV epidemic in South Africa only peaked in the late 1990s, and LE would have declined for another 10 years if ART were not rolled out in 2004.^22^ This is an important difference with a study from rural Uganda where LE gains of a similar magnitude have been registered.^6^ In the Ugandan case, mortality declines brought about by the rollout of ART are reinforced by mortality reductions due to earlier declines in HIV incidence, and the LE gain attributable to ART is smaller than the observed increase since ART. ^6,23^

The mortality reductions in the study population are large, but they did not come about immediately. Instead, the benefits of ART gradually unfolded, possibly in accordance with lowering ART eligibility thresholds, the availability of more efficacious treatment regimens, the rollout to primary healthcare facilities, and improvements in patients' engagement with HIV services. A few studies have indeed started to document earlier treatment initiation and better patient retention,^24,25^ and suggest that the early assessments of poor engagement with HIV services in generalized epidemics no longer apply.^26,27^ The gradual progress, however, raises the question of why health systems have not been able to capitalize on the benefits of highly effective treatment faster; why it has taken over a decade to reduce the burden of HIV on adult mortality to its current level. In addition, the residual burden of HIV on adult mortality is still not negligible, particularly among women whose adult LE in 2014 is still 5·3 (95%-CI: 2·6-7·8) years lower than that of HIV negative women. Among men, the LE deficit is estimated at 1·2 years and no longer statistically different from zero (95%-CI: -2·9-5·8). The decomposition of the LE deficit by cause affirms that pulmonary TB and HIV almost exclusively account for the surplus mortality, and an earlier analysis suggests that the majority of deaths to PLHIV in this population occur among those who have yet to start treatment.^9^ Pre-treatment mortality among PLHIV is, however, declining rapidly,^1^ and it will be important to continue monitoring mortality in relation to the cascade of HIV care and treatment to further improve the delivery of services.

Estimates for the LE deficit also highlight the dual nature of the gender disparities in HIV-associated mortality because the burden of HIV on adult mortality remains larger for women than for men, despite their more sizable gains in adult life-years to date. Several studies have suggested that women disproportionally benefit from the rollout of HIV care and treatment services in African populations, as indicated higher HIV testing and ART coverage rates,^28-30^ earlier treatment initiation, and lower attrition and mortality rates on ART.^30-33^ These conclusions are supported by our findings, but it is important to understand that the disproportionate burden of HIV on women, as quantified by the LE deficit, has not yet been fully rectified. We also need to appreciate that gender differences in LE gains and deficits do not solely result from differences in HIV services utilisation. Because women's HIV prevalence is higher than that of men, women lost more life-years to HIV as the epidemic unfolded and, consequently, had more life-years to gain from the expansion of treatment. In addition, women are infected at younger ages,^34^ and have lower mortality from causes unrelated to HIV. In the absence of treatment, a female HIV infection resulting in an early death will therefore incur a larger loss in life-years than a male HIV death. Conversely, preventing a female HIV death will result in a larger gain in life-years.

Another noteworthy gender difference relates to the age profile of HIV-associated mortality. As treatment programs matured, the median age of the LE deficit among women increased by 1·4 years, which indicates that the burden of HIV is not only declining, but also shifting to older ages. We do not currently observe this phenomenon among men, and that suggests that they still die in large numbers before or shortly after starting therapy.

The survival of PLHIV into older ages will inevitably complicate the attribution of CoDs among PLHIV because comorbidities are common.^35,36^ More generally, any assessment of changes in the CoD structure comes with the caveat that VAs are a relatively crude instrument for cause of death assignment and its specificity for identifying AIDS deaths remains unknown.^37^ However, our results indicate that the misclassification of causes is limited given that VAs attribute between 80 and 86% of the LE deficit to pulmonary TB and HIV, and much of the remainder to other communicable diseases. In other words, there is a close correspondence between the VA assigned causes of death and the mortality surplus in comparison to the population with a known HIV negatives status.

A final word of caution has to do with the attribution of the mortality gains to ART. Because the LE of HIV negative men and women has not changed over the period under observation, the LE gains must come from mortality reductions among PLHIV. The rollout ART is the most plausible reason for that, but we cannot exclude mortality reductions associated with improvements in TB treatment and programmes since TB and HIV are so difficult to distinguish. Similarly, PLHIV may benefit in other ways from their engagement with health services, but little is known about the existence and magnitude such spill-over effects.

## Supplementary Material

2

## Figures and Tables

**Table 1:** Characteristics of the study population, and death rates before and after the introduction of the ART (uMkhanyakude, 2001-'14)

	Individuals^a^	Person-Years	Deaths	2001-2004 Rate (95%-CI)	2011-2014 Rate (95%-CI)
All	93,903	535,428	9,992	23·1 (22·3-23-9)	13·6(13·0-14·2)
**Men**					
15-19	20,887	62,552	129	2 (1·5-2·8)	1·3 (0·9-2·0)
20-24	16,836	38,642	186	6·4(5·0-8·1)	3·1(2·2-4·3)
25-34	14,507	44,791	929	30·3 (27·3-33·6)	12·7(11·0-14·6)
35-44	8,171	28,863	969	45·4(41·1-50·2)	21·5 (18·6-24·9)
45+	7,799	46,576	2,432	59·2(55·1-63·5)	43·2 (39·8-46·8)
All men	42,262	221,424	4,645	26 (24·8-27·3)	15·6 (14·6-16·5)
**Women**					
15-19	21,736	61,327	136	2·7 (2·0-3·5)	1·5 (1·0-2·2)
20-24	19,158	44,946	353	11·1(9·4-13·1)	4·4 (3·4-5·7)
25-34	18,809	63,162	1,160	28·7 (26·3-31·3)	8·5 (7·2-9·9)
35-44	11,336	47,542	835	22·9 (20·5-25·5)	10·1(8·5-12·0)
45+	12,812	97,029	2,863	32·8(30·6-35·1)	24·6 (22·9-26·5)
All women	51,641	314,005	5,347	21.0 (20·1-22·0)	12·2(11·5-13·0)
**HIV status**^b^					
Negative	31,520	132,482	1,530	-	13·9 (12·9-14·9)^c^
Positive	15,148	59,576	2,142	-	23·4(21·8-25·2)
Unknown	93,253	343,370	6,320	23·4 (22·7-24·2)	9·2 (8·5-9·9)

Notes:

a Individuals can contribute to more than one category as they age or their HIV status changes during follow-up.

b We report the HIV status information as it is known to the study and may not be the same as men and women's knowledge of their own HIV status. Unknown HIV status includes all the persons-years of exposure before the start of the HIV surveillance as well as individual time prior to the first HIV test, and exposure time more than five years after the last HIV negative test.

c The death rate for adult HIV negatives is higher than for the population as a whole, which is due to the older age distribution of the population with a known HIV negative status (appendix, pp. 2-3).

**Table 2:** The contribution of cause of death groups to the LE gains and deficits (uMkhanyakude, 2001-'14)

	Men		Women	
	Years	%^a^	Years	%^a^
**LE gains: 2001-'04 to 2001-'14**				

HIV/AIDS	4·22	39·9	9·20	65·4
Pulmonary TB	4·21	39·8	3·56	25·3
Other CD	0·67	6·4	-0·32	-
Neoplasms	011	10	0·10	0·7
CVD	0·21	20	0·33	2·3
Other NCD	0·15	1·4	0·41	2·9
External	101	9·5	0·35	2·5
Maternal	-	-	0·12	0·9

**LE deficit: 2007-'10**				

HIV/AIDS	2·73	29·7	6·32	47·4
Pulmonary TB	507	55·3	508	38·1
Other CD	1·16	12·6	0·86	6·4
Neoplasms	-0 06	-	009	0·6
CVD	-008	-	0·14	1·1
Other NCD	-0·18	-	0·68	5·1
External	0·21	2·3	0·10	0·8
Maternal	-	-	006	0·5

**LE deficit: 2011-'14**				

HIV/AIDS	1·79	27·4	3·92	44·5
Pulmonary TB	3·75	57·5	3·20	36·3
Other CD	0·67	10·3	1·27	14·4
Neoplasms	004	0·6	0·26	2·9
CVD	-0·21	-	-0 02	-
Other NCD	-0·50	-	-0·36	-
External	0·27	4·7	003	0·3
Maternal	-	-	0·13	1·5

Notes:

a Percent of the sum of positive differences in adult LE.

TB=tuberculosis, CD=communicable diseases, CVD= cardiovascular disease, NCD=non-communicable disease

## Research in Context

### Evidence before this study

We searched PubMed and Medline in June 2016 for studies on the impact of ART and the residual burden of HIV on adult mortality. We did not apply any language or date restrictions, and used combinations of the search terms “HIV”, “AIDS”, “life expectancy”, “population”, “antiretroviral therapy” and “burden”. Several studies report on the life expectancy of HIV positive individuals who started ART, and a number of institutions regularly report on mortality estimates that are the result of more complex modelling exercises. Two studies, one from Uganda and one from South Africa, provide direct nonparametric estimates of population-wide changes in (adult) life expectancy following the rollout of antiretroviral therapy in generalized HIV epidemics. The South African study is based on the same data source used here, and reported an increase of 11·3 years (both sexes) in the in the life expectancy at age 15 between 2003, the year before ART was rolled out, and 2011.

### Added value of this study

We extend the analyses for KwaZulu-Natal to 2014, and document per capita adult life expectancy gains of 1·38 years per annum for men and 1·58 years for women, for a total gain since ART of 15·2 (95%-CI: 12·4-17·8) and 17·2 years (95%-CI: 14·5-20·2) for men and women, respectively. We expand on these findings in two novel ways. First, we quantify the residual burden of HIV-associated mortality as the shortfall or deficit of the population-wide life expectancy compared to the life expectancy of the HIV negative population. Among others, this demonstrates that the remaining burden of HIV has become relatively small, especially among men. Among women, the adult life expectancy deficit is still 5·3 years (95%-CI: 2·6-7·8). Second, we use verbal autopsy (VA) data and a new VA interpretation tool (InSilicoVA) to establish that differences in mortality from pulmonary TB and HIV explain most of the recent gains in adult life expectancy as well as the remaining life expectancy deficit.

### Implications of all the available evidence

Unprecedented increases in adult life expectancy associated with a reduction in HIV-related mortality underscore the success of the ART programme in this population. The burden of HIV mortality remains, however, sizable for women, despite their better engagement with HIV care services. Women, who have to date gained more adult life-years than men, continue to bear the highest burden of HIV mortality, which is a finding that adds a new perspective to the literature wherein men are often portrayed as the “*losers of the ART scale-up.*”
