# Supplementary material for: The burden of HIV mortality following the rollout of antiretroviral therapy: evidence from an observational community cohort study in KwaZulu-Natal, South Africa
Source: Lancet HIV. Author manuscript; Available in PMC 2018 Mar 1. (PMC5405557; doi:10.1016/S2352-3018(16)30225-9)
Supplement: 2 [file NIHMS836858-supplement-2.pdf]

THE LANCET HIV

Supplementary appendix

This appendix formed part of the original submission and has been peer reviewed. We post it as supplied by the authors.

Supplement to: Reniers G, Blom S, Calvert C, et al. Trends in the burden of HIV mortality after roll-out of antiretroviral therapy in KwaZulu-Natal, South Africa: an observational community cohort study. *Lancet HIV* 2016; published online Dec 9. http://dx.doi.org/10.1016/S2352-3018(16)30225-9.

Trends in the burden of HIV mortality after rollout of antiretroviral therapy in KwaZulu-Natal, South Africa: an observational community cohort study – appendix

G. Reniers, PhD [1,2] S. Blom, MSc [1] C. Calvert, PhD [1] A. Martin-Onraet, MD [3] A.J. Herbst, MSc(Med) [4] J.W. Eaton, PhD [5] J. Bor, ScD [6] E. Slaymaker, PhD [1] Z.R. Li, MA [7] Prof. S.J. Clark, PhD [2,8] Prof. T. Bärnighausen, MD [4,9,10] Prof. B. Zaba, MSc [1] and Prof. V. Hosegood PhD[4,11]

[1] Department of Population Health, London School of Hygiene and Tropical Medicine, London, United Kingdom
[2] School of Public Health, University of the Witwatersrand, Johannesburg, South Africa
[3] Instituto Nacional de Cancerología, Mexico City, Mexico
[4] Africa Health Research Institute, Durban, South Africa
[5] Department of Infectious Disease Epidemiology, Imperial College, London, United Kingdom
[6] Department of Global Health, Boston University, Boston, United States
[7] Department of Statistics, University of Washington, Seattle, United States
[8] Department of Sociology, The Ohio State University, Columbus, Ohio
[9] Institute of Public Health, University of Heidelberg, Heidelberg, Germany
[10] Harvard T.H. Chan School of Public Health, Harvard University, Boston, United States
[11] Social Statistics and Demography, University of Southampton, Southampton, United Kingdom

1. HIV status information in the dataset

HIV status information comes from HIV seroprevalence surveys that are conducted annually since 2003-2004. Initially only men and women of reproductive age were invited to participate in the serosurveys; in 2007, the age-eligibility criteria were expanded to all adults. This is complemented with HIV status information retrieved via record linkage between the HDSS and HIV care and treatment facilities.[1] The latter only identifies HIV positive men and women.

The allocation of exposure time to the various HIV status categories is done in the following manner: person-time before the first HIV test is treated as HIV status unknown, and the time following an HIV positive test is treated as HIV positive exposure time. Analyses of mortality by HIV status also require an assumption about the amount of time that individuals remain HIV negative following an HIV-negative test. In this study, we assume that persons remain HIV negative for five years after their last negative HIV test, after which their HIV status is reclassified as unknown. To test the suitability of this assumption, we calculated the probability that deaths classified as HIV negative should have been treated as HIV positive deaths because a person could seroconvert and die from an HIV related cause in the five-year window after the last HIV negative test. For each death, this calculation relied on (i) the actual duration between an individual's last HIV negative test date and date of death, (ii) the observed sex/age-specific HIV incidence rate, (iii) HIV survival as a function of age at seroconversion,[2] (iv) the effects of ART on HIV mortality by calendar period, and (v) the observed sex and age-specific non-HIV mortality rates.

Table A1: Duration from last negative HIV test to death and probability of death misclassification by sex, 2010-2014

	Women			Men		
**Age group**	**Number of HIV-deaths[a]**	**Mean (IQR) years last neg. to death**	**Avg. misclassification probability (95% CI)[b]**	**Number of HIV-deaths[a]**	**Mean (IQR) years last neg. to death**	**Avg. misclassification probability (95% CI)[b]**
15-24 years	17	1·9 (0·7-2·8)	0·034 (0·026-0·043)	27	2·1 (0·9-3·6)	0·014 (0·010-0·020)
25-34 years	14	1·9 (0·9-2·9)	0·043 (0·032-0·058)	25	2·5 (0·8-3·6)	0·036 (0·026-0·048)
35-44 years	14	2·0 (0·7-3·5)	0·028 (0·020-0·037)	20	1·6 (0·7-2·3)	0·007 (0·004-0·010)
45-54 years	32	1·5 (0·8-2·4)	0·003 (0·002-0·004)	38	1·7 (0·6-2·3)	0·004 (0·003-0·006)
55-64 years	64	1·4 (0·6-1·9)	0·001 (0·000-0·001)	78	1·8 (0·7-2·7)	0·001 (0·000-0·001)
65-74 years	134	1·7 (0·7-2·5)	<0·001	106	1·3 (0·5-2·1)	<0·001
75-84 years	179	1·6 (0·7-2·3)	<0·001	80	1·7 (0·8-2·2)	<0·001
85+ years	70	1·9 (0·8-2·9)	<0·001	50	1·4 (0·7-2·0)	<0·001
Total	524	1·6 (0·6-2·4)	0·003 (0·003 0·004)	424	1·7 (0·7-2·5)	0·004 (0·003-0·005)

Notes: [a] Number of deaths classified as being to HIV-negative adults in analyses stratified by HIV status, based on deaths that occurred within 5 years of the individuals' last negative HIV test. [b] The average probability that an individual seroconverted and died from HIV in the interval between the last HIV test and date of death. The complement (1 – probability) is the average probability that the death was truly not related to HIV.

Table A1 reports the number of deaths classified to HIV-negative adults (within 5 years of the last HIV-negative test), the mean duration from the last HIV-negative test to the observed death, and the average probability that a death was attributable to HIV. This exercise illustrates that the probability of misclassifying deaths remains under 5% for all age groups and sexes. The probability of misclassification was highest for the 15-35 year age group in which HIV incidence is highest and non-HIV mortality rates are lowest. In older age groups, the probability that HIV-negative deaths were misclassified is vanishingly small (<1%).

Table A2 illustrates that the coverage of the HIV status information in the dataset gradually increased since the start of the population-based HIV serosurveys. HIV status information remains under 60% in the most recent years, however, and that is due to the relatively low HIV survey participation rates.[3]

It is also worth noting that ACDIS tracks the vital status of both resident and non-resident household members, but we only include residents in our analyses because the ascertainment of HIV status and causes of death (CoD) is more complete for residents.

Table A2: Coverage of the HIV status information in the dataset by calendar year and sex (percent of person years)

Year	Men	Women	Both sexes
2000	0·0	0·0	0·0
2001	0·0	0·0	0·0
2002	0·1	0·1	0·1
2003	3·7	4·2	4·0
2004	21·2	21·9	21·6
2005	32·0	32·0	32·0
2006	36·9	36·3	36·6
2007	39·1	42·6	41·2
2008	42·5	51·1	47·6
2009	41·9	53·6	48·8
2010	40·9	55·9	49·6
2011	42·6	59·9	52·7
2012	42·6	60·4	52·9
2013	44·9	63·0	55·4
2014	45·7	64·9	56·9

The relative age distribution of the population by HIV status and sex is illustrated in Figure A1. As one would expect, most PLHIV are between 20 and 60 years old. The age profile of the HIV negative population is older than that of the population as a whole, which helps to explain the higher crude death rate (ages 15 and above) among the HIV negative population reported in Table 1 in the article.

Figure A1: One-year age densities by HIV status and sex (uMkhanyakude, July 2014)

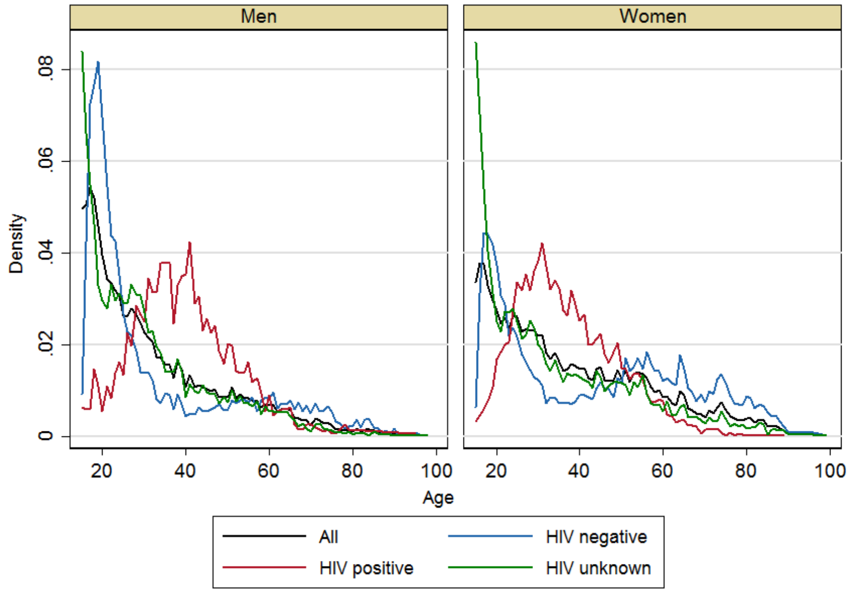

2. Adult life expectancy (LE) estimates and their interpretation

Table A3 contains the adult LE estimates that are the basis for Figure 1 in the main article. The adult LE is estimated with non-parametric survival analysis methods as the area under the Kaplan-Meier survival curve. Percentile-based confidence intervals are obtained via bootstrapping with 1,000 replications. A similar approach has been taken elsewhere.[4,5] Because of the sparse data in old age and to avoid possible bias due to age over-reporting, we truncate the person-years lived above age 100. The precise interpretation of our LE measure is the expected number of years lived between ages 15 and 100 given the age-period mortality rates. On average, the difference with the LE at age 15 is less than 0.1 years, but it is slightly larger for women than for men. Adding 15 years to our estimates gives the mean age at death conditional on survival to age 15. The latter is the measure reported by Bor and colleagues in other studies based on the same data source.[4,6]

A key attribute of adult LE as a metric for studying HIV mortality and the impact of ART is that it quantifies the postponement rather than the elimination of deaths. Measures that do not account for the shift in the age distribution of mortality (e.g., the number of AIDS deaths, death rates or the probability of dying in adulthood) are less sensitive to the nature of the mortality reductions induced by ART and will underestimate its impact. Another matter of interpretation that affects any synthetic mortality measure is that the LE may be inflated in the period that the bulk of HIV positives initiate treatment. In those years, deaths among HIV positives are averted, but their elevated mortality at older ages does not yet factor into the estimates. This phenomenon invalidates the interpretation of the synthetic or period LE as an estimate of the years lived *under current conditions*, but it remains an accurate measure of the years lived *at current rates.*[7]

Table A3: Non-parametric adult life expectancy estimates by sex, HIV status and calendar year, uMkhanyakude (2001-2014)

				**Men**				
	**2001**	**2003**	**2005**	**2007**	**2009**	**2011**	**2013**	**2014**
LE All	31·4	30·8	33·1	33·6	36·6	40·4	41·3	45·9
	(29·9-33·1)	(29·2-32·5)	(31·2-35·0)	(31·9-35·5)	(34·8-38·5)	(38·5-42·5)	(39·1-43·3)	(43·7-48·4)
LE HIV-	-	-	-	47·4	44·4	48·1	46·1	47·0
				(43·4-51·9)	(40·6-48·6)	(43·9-52·7)	(41·7-50·7)	(42·5-51·7)
LE HIV+	-	-	-	12·1	17·8	25·3	33·1	30·5
				(0·0-20·6)	(3·9-27·1)	(21·1-29·4)	(28·4-39·3)	(24·4-38·3)
LE HIV unk.	-	-	-	35·8	40·9	44·7	44·1	51·6
				(33·5-38·5)	(37·7-44·4)	(41·7-48·0)	(40·9-47·6)	(48·4-55·4)
LE deficit	-	-	-	13·8	7·8	7·8	4·8	1·2
				(9·7-18·6)	(3·8-11·5)	(4·2-12·0)	(0·7-9·2)	(-2·9-5·8)
				**Women**				
	**2001**	**2003**	**2005**	**2007**	**2009**	**2011**	**2013**	**2014**
LE All	40·7	37·0	38·7	41·5	46·3	50·2	53·6	54·2
	(38·6-42·8)	(35·2-38·9)	(36·8-40·7)	(39·4-43·4)	(44·3-48·5)	(48·1-52·7)	(51·3-55·8)	(52·2-56·2)
LE HIV-	-	-	-	59·2	58·1	60·7	62·6	59·4
				(54·1-65·0)	(54·5-61·8)	(57·2-64·0)	(59·5-65·5)	(56·3-62·3)
LE HIV+	-	-	-	23·4	29·2	31·6	38·9	44·1
				(19·7-26·2)	(25·8-32·6)	(23·3-37·9)	(35·6-42·9)	(39·1-52·1)
LE HIV unk.	-	-	-	45·8	51·4	56·1	57·0	56·4
				(43·0-48·9)	(48·4-54·4)	(52·2-59·9)	(52·9-61·0)	(52·8-60·7)
LE deficit	-	-	-	17·7	11·8	10·4	9·0	5·3
				(12·3-23·8)	(8·2-15·1)	(7·5-13·3)	(6·4-11·9)	(2·6-7·8)

Aside from the overall adult LE, we also report the LE by HIV status and the adult LE deficit. The LE of HIV negative adults is a measure of the background or HIV free mortality, and is an important indicator of the health profile of a population in its own right. In our case, it also serves as a benchmark of the achievable LE in the population that we use to calculate the LE deficit (see below). The LE estimates for the HIV negative population are around 47 years for men and 60 years for women. In comparison, Bor and colleagues report an HIV and TB cause-deleted adult LE of 50 years for men, and 60 years for women.[4]

The LE of HIV positive men and women quantifies the number of additional years that an HIV positive 15-year old can expect to live at current rates, and is of direct interest to any study evaluating the impact of ART. Because of the relatively small numbers, these estimates have wide uncertainty bounds. In addition, trends in the LE of PLHIV could be biased when the interval between seroconversion and the time that PLHIV become known HIV positives to the study varies.

The LE for people whose HIV status is unknown is informative about possible selection with respect to the HIV status information in the datasets. In this case, the LE of men and women with an unknown HIV status is a little higher than that of the population as a whole, which suggests that the group of residents with an unknown HIV status contains a disproportionally large share of HIV negatives. This apparently contradicts studies that have demonstrated that PLHIV are less likely to participate in HIV serological surveys,[8-12] but is explained by the fact that we also identify PLHIV via record linkage with medical facilities that provide HIV care and treatment services.

The adult LE deficit is the difference between the adult LE of known HIV negatives and the adult LE for the population as a whole. It is, in other words, a summary measure of the mortality burden of HIV conditional on the background health profile of its population. The LE deficit is directly affected by HIV epidemic severity and efforts to mitigate its mortality impact (e.g., ART). Not all of the LE deficit is necessarily attributable to HIV, however, as the LE deficit could be inflated due to a positive correlation between HIV infection and non-AIDS mortality risks factors including socio-economic background, smoking and substance abuse. To date, most evidence of such an association comes from high income settings and concentrated epidemics.[13] Similarly, the expansion of treatment programs could generate spill-over effects on non-AIDS mortality of both HIV negatives and HIV positives via changes to health services provision or health seeking behaviour, a decline in suicide rates, and so forth.[4,14] Little is known about the magnitude of such spill-over effects. When interpreting differences in the LE deficit between population subgroups, it is also important to understand that the LE deficit is sensitive to variability in the age at infection and differences in background mortality. Finally, we also want to point at the distinction between the LE deficit as defined above, and studies that report the LE of HIV positive men and women as a percentage of the LE of HIV negatives or the population as a whole.[15,16] The latter quantify the shortfall in the LE of PLHIV, but do not measure the population-wide burden of HIV.

3. Cause of death classification

Table A4 maps the CoD classification scheme used in the manuscript onto their respective ICD-10 codes.

Table A4: Cause of death (CoD) classification

CoD groups in manuscript	ICD-10 code
HIV/AIDS related	B20-B24
Pulmonary tuberculosis	A15-A16
Other communicable diseases & nutritional conditions	A00-A09; A17-A99; B00-B19; B25-B99; D50-D64; E40-E46; G00-G05; J00-J22
Maternal disorders	O00-O08; O10-O16; O20-O99
Malignant neoplasms	C00—C26; C30-C58; C60-D48
Cardiovascular diseases	D57; I00-I15; I20-I52; I60- I99
Other non-communicable diseases	D55-D89; E00-E07; E10-E35; E50-E90; F00-F99; G06—G37; G40-G41; G50-G99; H00-H95; J30-J99; K00-K31; K35-K38; K40-K93; L00-L99; M00-M99; N00-N99; R00-R94
External injuries	S00-T99; V01-V99; W00-W99; X00-X99; Y00-Y98
Indeterminate	R95-R99

4. Decomposition of LE differences based on the InterVA model for cause of death attribution

Table A5 summarizes the decomposition of adult LE differences based on CSMFs estimated with the R package of the InterVA-4 model (version 1.5) with the replicate option set to false.[17,18] InterVA-4 generates up to three probable CoDs with their corresponding likelihoods or an indeterminate CoD with 100% likelihood. If the likelihoods of the first three CoDs do not sum to 100%, the residual is treated as indeterminate. As with InSilicoVA, CoDs that were classified as missing due to incomplete VAs were excluded from the analyses.

CoD attribution was done separately by HIV status to minimize misclassification of HIV deaths. InterVA is run with malaria prevalence set to low and HIV prevalence set to high for known HIV positives and for men and women whose HIV status is unknown, and with both malaria and HIV prevalence set to low for the HIV negative population. Unlike InSilicoVA, InterVA allows for deaths to be classified as indeterminate, but their contribution to the LE differences are small. The only other noteworthy difference between the two VA interpretation tools for our analyses is that InSilicoVA tends to attribute a slightly higher fraction of the LE differences to other (unspecified) communicable diseases.

Table A5: The CoD contributions to the LE gain and deficits based on the CoD attribution with the InterVA model (uMkhanyakude, 2001-‘14)

	**Men**		**Women**	
	**Years**	**% of total[a]**	**Years**	**% of total[a]**
**LE gain: 2001-'04 to 2001-'14**				
HIV/AIDS	3·71	35·1	7·72	55·3
TB	4·17	39·5	4·77	34·2
Other CD	0·37	3·5	0·10	0·7
Neoplasms	0·67	6·4	0·42	3·0
CVD	0·25	2·3	0·45	3·3
Other NCD	0·15	1·4	0·00	0·0
External	0·89	8·4	0·32	2·3
Maternal	-	-	0·16	1·2
Indeterminate	0·36	3·4	-0·22	-
**LE deficit: 2007-'10**				
HIV/AIDS	3·95	42·6	6·99	52·2
TB	4·16	44·8	4·24	31·7
Other CD	0·43	4·7	0·77	5·7
Neoplasms	0·49	5·3	0·39	2·9
CVD	-0·04	-	-0·07	-
Other NCD	-0·33	-	0·46	3·4
External	0·24	2·6	0·12	0·9
Maternal	-	-	0·07	0·5
Indeterminate	-0·06	-	0·36	2·7
**LE deficit: 2011-'14**				
HIV/AIDS	2·06	34·0	4·75	54·3
TB	3·68	60·7	2·57	29·5
Other CD	-0·08	-	0·49	5·6
Neoplasms	0·01	0·2	0·22	2·6
CVD	-0·17	-	-0·04	-
Other NCD	0·12	2·0	0·19	2·1
External	0·16	2·6	-0·07	-
Maternal	-	-	-0.19	-
Indeterminate	0.03	0.5	0.52	5.9

Notes: [a] Percent of the sum of positive differences in adult LE

5. Comparison of adult LE trends in uMkhanyakude and estimates for South Africa as a whole

Even though these are not directly comparable, it is useful to contrast our adult LE estimates for uMkhanyakude with two sets of estimates for South Africa as a whole: one from the United Nations Population Division's (UNPD) 2015 World Population Prospects,[19] and the second set of estimates from the Institute of Health Metrics and Evaluation's (IHME) 2013 Global Burden of Disease (GBD) study.[20] This is done in Figure A2.

Adult LE estimates from IHME and UNPD are comparable for men, but the GBD estimates entail larger gender differences. The trend in adult LE is quite different in uMkhanyakude. Because the HIV epidemic is particularly severe in this population (29.0% among adults of reproductive age in the study population versus 18.8% for South Africa as a whole), [21,22] one would indeed expect that adult LE bottoms out at a lower level and subsequently increases faster because mortality will fluctuate more drastically in a high prevalence population where the mortality impact of the epidemic first unfolds and is later mitigated by the rollout of ART. More surprising, however, is that the adult LE in uMkhanyakude is estimated to be as high as or even higher than the national-level estimates. It is difficult to adjudicate on the accuracy of each set of estimates on the basis of this evidence alone. Possible reasons for the discrepancy are (i) that adult mortality is underestimated in the HDSS, (ii) that the treatment programs are particularly effective in the facilities that serve the HDSS population, or, (iii) that the national-level adult LE estimates from the UNPD and IHME are too low, possibly because they underestimate the mortality reductions brought about by the rollout of ART.

Figure A2: Adult life expectancy trends in uMkhanyakude, and South Africa (RSA) as a whole, 1995-2014

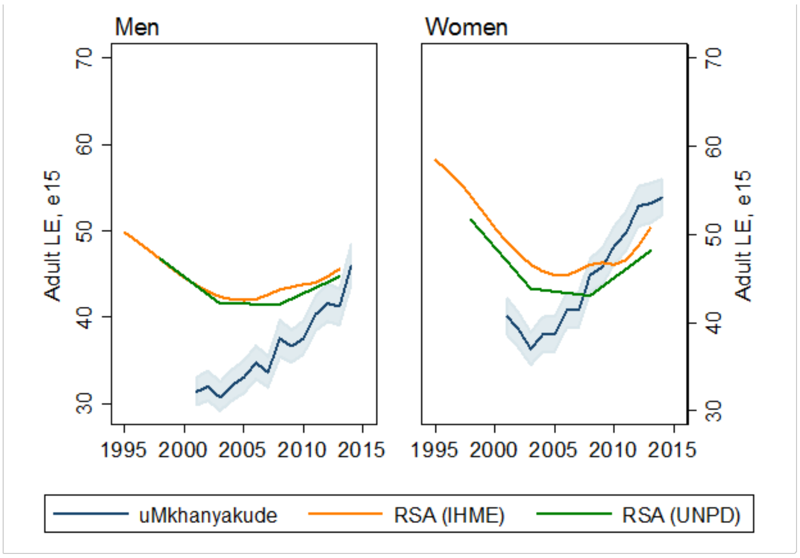

References

1. Barnighausen T, Tanser F, Malaza A, Herbst K, Newell ML. HIV status and participation in HIV surveillance in the era of antiretroviral treatment: a study of linked population-based and clinical data in rural South Africa. *Trop Med Int Health* 2012; **17**(8): e103-10.
2. Todd J, Glynn JR, Marston M, et al. Time from HIV seroconversion to death: a collaborative analysis of eight studies in six low and middle-income countries before highly active antiretroviral therapy. *AIDS* 2007; **21**: S55-S63.
3. Tanser F, Hosegood V, Bärnighausen T, et al. Cohort Profile: Africa centre demographic information system (ACDIS) and population-based HIV survey. *Int J Epidemiol* 2008; **37**(5): 956-62.
4. Bor J, Herbst AJ, Newell M-L, Bärnighausen T. Increases in adult life expectancy in rural South Africa: valuing the scale-up of HIV treatment. *Science* 2013; **339**(6122): 961-5.
5. Asiki G, Reniers G, Newton R, et al. Adult life expectancy trends in the era of antiretroviral treatment in rural Uganda (1991-2012). *AIDS* 2016; **30**(3): 487-93.
6. Bor J, Rosen S, Chimbindi N, et al. Mass HIV Treatment and Sex Disparities in Life Expectancy: Demographic Surveillance in Rural South Africa. *PLoS Med* 2015; **12**(11): e1001905; discussion e.
7. Vaupel JW. Lifesaving, lifetimes and lifetables. How Long Do We Live?: Springer; 2008: 93-107.
8. Reniers G, Eaton J. Refusal bias in HIV prevalence estimates from nationally representative seroprevalence surveys. *AIDS* 2009; **23**(5): 621-9.
9. Barnighausen T, Bor J, Wandira-Kazibwe S, Canning D. Correcting HIV prevalence estimates for survey nonparticipation using Heckman-type selection models. *Epidemiology* 2011; **22**(1): 27-35.
10. Floyd S, Molesworth A, Dube A, et al. Underestimation of HIV prevalence in surveys when some people already know their status, and ways to reduce the bias. *AIDS* 2013; **27**(2): 233-42.
11. Nyirenda M, Hosegood V, Barnighausen T, Newell ML. Mortality levels and trends by HIV serostatus in rural South Africa. *AIDS* 2007; **21 Suppl 6**: S73-9.
12. Larmarange J, Mossong J, Barnighausen T, Newell ML. Participation dynamics in population-based longitudinal HIV surveillance in rural South Africa. *PLoS One* 2015; **10**(4): e0123345.
13. Sabin CA. Do people with HIV infection have a normal life expectancy in the era of combination antiretroviral therapy? *BMC medicine* 2013; **11**: 251.
14. Hontelez JA, Tanser FC, Naidu KK, Pillay D, Barnighausen T. The Effect of Antiretroviral Treatment on Health Care Utilization in Rural South Africa: A Population-Based Cohort Study. *PLoS One* 2016; **11**(7): e0158015.
15. Wandeler G, Johnson LF, Egger M. Trends in life expectancy of HIV-positive adults on antiretroviral therapy across the globe: comparisons with general population. *Curr Opin HIV AIDS* 2016; **11**(5): 492-500.
16. Johnson LF, Mossong J, Dorrington RE, et al. Life expectancies of South African adults starting antiretroviral treatment: collaborative analysis of cohort studies. *PLoS Med* 2013; **10**(4): e1001418.
17. Zehang L, McCormick T, Clark S. InterVA4: An R package to analyze verbal autopsy data. In: University of Washington, editor. CSSS Working Paper; 2014.
18. Byass P, Chandramohan D, Clark SJ, et al. Strengthening standardised interpretation of verbal autopsy data: the new InterVA-4 tool. *Glob Health Action* 2012; **5**: 1-8.
19. UNPD. World Population Prospects: The 2015 Revision 2015. https://esa.un.org/unpd/wpp/Download/Standard/Mortality/ (accessed 20 September 2016).
20. IHME. Global Burden of Disease Study 2013: Age-Sex Specific All-Cause and Cause-Specific Mortality 1990-2013. 2014. http://ghdx.healthdata.org/global-burden-disease-study-2013-gbd-2013-data-downloads (accessed 20 September 2016).
21. Zaidi J, Grapsa E, Tanser F, Newell ML, Barnighausen T. Dramatic increase in HIV prevalence after scale-up of antiretroviral treatment. *AIDS* 2013; **27**(14): 2301-5.
22. Shisana O, Labadarios D, Simbayi L, et al. South African national HIV prevalence, incidence and behaviour survey, 2012. 2015.
